# Autoantibodies and Resident Renal Cells in the Pathogenesis of Lupus Nephritis : Getting to Know the Unknown

**DOI:** 10.1155/2012/139365

**Published:** 2012-06-14

**Authors:** Susan Yung, Tak Mao Chan

**Affiliations:** Department of Medicine, Queen Mary Hospital, University of Hong Kong, Pokfulam, Hong Kong

## Abstract

Systemic lupus erythematosus is characterized by a breakdown of self-tolerance and production of autoantibodies. Kidney involvement (i.e., lupus nephritis) is both common and severe and can result in permanent damage within the glomerular, vascular, and tubulo-interstitial compartments of the kidney, leading to acute or chronic renal failure. Accumulating evidence shows that anti-dsDNA antibodies play a critical role in the pathogenesis of lupus nephritis through their binding to cell surface proteins of resident kidney cells, thereby triggering the downstream activation of signaling pathways and the release of mediators of inflammation and fibrosis. This paper describes the mechanisms through which autoantibodies interact with resident renal cells and how this interaction plays a part in disease pathogenesis that ultimately leads to structural and functional alterations in lupus nephritis.

## 1. Introduction

Lupus nephritis is a severe organ manifestation of systemic lupus erythematosus (SLE) that can affect up to 70% of the SLE population [1]. Depending on the severity of disease, 10–30% of these patients will progress to end-stage renal failure. Lupus nephritis is characterized by the production of anti-double-stranded (ds) DNA antibodies and immune-mediated injury in the glomerular, vascular, and tubulo-interstitial compartments of the kidney [2–9]. If left untreated, destruction of the normal renal parenchyma and their replacement with fibrous tissue ensues [7]. Lupus nephritis follows a relapsing-remitting pattern in which the frequency of flares differs between individual patients. Clinical manifestations of active lupus nephritis include proteinuria, active urinary sediments, and progressive renal dysfunction [10, 11].

Anti-dsDNA antibodies have been shown to contribute to the pathogenesis of lupus nephritis. Many features of lupus nephritis can be replicated in nonautoimmune mice after either intraperitoneal administration of human or murine anti-dsDNA antibodies or inoculation with the transgene that encodes the secreted form of an IgG anti-DNA antibody [12, 13]. It has remained intriguing how these antibodies deposit in the kidneys and trigger intrarenal pathogenic mechanisms. Various mechanisms of antibody binding have been proposed, some of which remain controversial. The origin of anti-dsDNA antibodies, their pathogenic role, and the characteristics associated with nephritogenic property have been extensively studied in experimental and *in vitro* systems [2, 3, 6, 13–15]. The data to date shows that polyreactivity and the ability to interact with various cell surface, intracellular, or extracellular molecules could be a pivotal property that allows the antibodies to elicit injury in the kidney [16–19]. This paper will discuss the contributing roles of resident renal cells in the pathogenesis of lupus nephritis through their interaction with anti-dsDNA antibodies, thereby inducing inflammatory and fibrotic processes in the kidney. Mechanisms through which lymphocytes and macrophages contribute to the pathogenesis of lupus nephritis have been discussed in recent papers [20–22].

### 1.1. Anti-dsDNA Antibodies and Lupus Nephritis

Production of autoantibodies is a cardinal feature of SLE [23]. The production of antibodies towards chromatin material, in particular to dsDNA, is strongly associated clinically with lupus nephritis [4–6, 23–28]. Other autoantibodies have also been described in patients with lupus nephritis [18, 29–38] and these are listed in Table 1.

Anti-DNA antibodies constitute a subgroup of anti-nuclear antibodies that bind to either single-stranded or double-standard DNA [2]. These antibodies form part of the normal spectrum of natural antibodies in healthy individuals which are predominantly of the IgM class and react weakly with self-antigens. In lupus patients, these “natural” antibodies undergo an isotype switch to IgG that increases their pathogenic potential [2]. Somatic mutations in the encoding immunoglobulin genes can also result in the secretion of high-affinity IgG anti-dsDNA antibodies [2, 39]. It is this subset of anti-dsDNA antibodies that have been implicated in pathogenesis of SLE and glomerulonephritis. Anti-dsDNA antibodies of the IgG subclass, in particular those of the IgG_1 _ and IgG_3_ subclass which can fix complement, are important in pathogenesis and also as a disease biomarker [2, 40, 41]. Anti-dsDNA antibodies have been detected in the sera of SLE patients before clinical onset of disease [42], and the prevalence of anti-dsDNA antibodies in patients with lupus nephritis is 70–96% compared to 0.5% in patients with nonlupus autoimmune disease or in healthy subjects [29, 31, 43]. Other factors that determine the nephritogenicity of anti-DNA antibodies include avidity of antigen binding, charge, and amino acid sequence in the complementarity determining region, as reviewed by Foster et al. [8] and Isenberg et al. [27].

Circulating levels of anti-dsDNA antibodies correlate with disease activity in many patients [4, 24, 27, 44]. Winfield et al. demonstrated that the affinity of circulating anti-dsDNA antibodies to dsDNA correlated with the activity of nephritis [14]. They also noted that the anti-dsDNA activity in IgG fractions eluted from nephritic glomeruli was higher than that in corresponding serum samples [14].

## 2. Mechanisms through Which Lupus Autoantibodies Mediate Kidney Injury

Onset of lupus nephritis is initiated by the deposition of anti-dsDNA antibodies in the renal parenchyma. The exact mechanism through which anti-dsDNA antibodies are deposited in the kidney to mediate kidney injury remains to be fully elucidated. Three mechanisms have been proposed, and they include (1) the deposition of preformed circulating DNA/anti-dsDNA immune complexes in the kidney, (2) binding of antibodies to antigens deposited within the kidney—the “planted antigen” theory, and (3) direct binding to cross-reactive antigens present either on the surface of resident renal cells or in their extracellular environment.

### 2.1. Entrapment of Circulating Preformed DNA/Anti-dsDNA Immune Complexes

It had been postulated that renal injury in lupus patients was due to the passive entrapment of circulating preformed DNA/anti-dsDNA immune complexes in the glomerulus. This theory has now been disproved since preformed immune complexes are difficult to detect in the blood, and studies have demonstrated that they are only transiently localized to the glomeruli before they are rapidly removed by the liver [45]. Following administration to nonautoimmune mice, preformed DNA/anti-dsDNA immune complexes have no affinity for components of the glomerular basement membrane (GBM) [46]. Furthermore, after administration of these immune complexes to lupus-prone mice, the level of anti-dsDNA antibodies and disease activity decreased [47].

### 2.2. “Planted Antigen” Theory

In the “planted antigen” theory, chromatin materials released into the circulation from apoptotic or necrotic cells are entrapped within the GBM where they serve as “planted antigens” to mediate binding of anti-dsDNA antibodies. Studies have suggested that the positively charged histone component of nucleosomes may initially bind to heparan sulfate proteoglycans in the GBM through charge-charge interactions, which exposes the DNA component of the nucleosome to act as a “planted antigen” or intermediate bridge for anti-dsDNA antibody binding [48, 49]. Subsequent studies have corroborated that anti-dsDNA antibodies can bind to the glomerulus through nucleosomes [26, 48–50]. Kramers et al. demonstrated that the perfusion of anti-dsDNA antibodies complexed to nucleosomal material into Wistar rats resulted in their deposition in the glomerular capillaries [51]. Subcutaneous administration of heparin to NZB/W F1 mice resulted in reduced nucleosome-containing immune complexes in the GBM and delayed development of disease manifestations suggesting that heparin may compete with extracellular heparan sulfate proteoglycans for nucleosome binding, thereby reducing immune complex formation in the kidney parenchyma [52]. Ultrastructural studies by Rekvig's group have demonstrated that anti-dsDNA antibodies colocalize with chromatin material in electron-dense deposits in the diseased kidney [26, 50]. The role of nucleosomes in the pathogenesis of lupus nephritis has been reviewed by Mortensen and Rekvig [53]. Anti-nucleosome antibodies have also been detected in SLE patients particularly in patients with renal flare [54]. Some have proposed that anti-nucleosome antibodies may be a disease biomarker for lupus nephritis [54, 55].

### 2.3. Cross-Reactivity with Non-DNA Antigens

Autoreactivity to native DNA *per se* does not appear to be a property of anti-dsDNA antibodies that are responsible for inducing renal injury. Immunization of non-autoimmune mice with mammalian DNA failed to induce the production of pathogenic anti-dsDNA antibodies or clinical manifestations of disease. Rather, there is emerging evidence that polyreactivity of anti-dsDNA antibodies, independent of chromatin material acting as a bridge for binding, confers pathogenic potential. Polyreactivity of anti-dsDNA antibodies may be related to structural or conformational similarity, or molecular mimicry [56]. Cross-reactivity of anti-dsDNA antibodies was first observed by Raz et al., who demonstrated that human and murine anti-DNA antibodies could bind directly to renal antigens in isolated rat kidneys, and this resulted in the induction of proteinuria [57]. Krishnan et al. demonstrated that anti-dsDNA antibodies from lupus-prone mice when injected intravenously into BALB/c mice could bind to the GBM and mesangial matrix and induce disease manifestations, and that these processes were independent of the binding of the antibodies to chromatin material [58]. Waters et al. observed that NZM congenic mice developed chronic glomerulonephritis in the absence of anti-dsDNA antibodies [59]. Christensen et al. also noted that nephritis developed in Toll-like receptor-9- (TLR-9) deficient lupus-prone mice despite the absence of anti-dsDNA antibodies [60]. It is thus possible that the reactivity of antibodies towards DNA or chromatin material *per se* may not be critical for the development of lupus nephritis, but rather the ability of autoantibodies to bind to various antigens in the renal parenchyma. *α*-actinin, heparan sulfate proteoglycan, laminin, fibronectin and collagen have been reported as putative antigens that are recognized by anti-dsDNA antibodies [16–18, 61–63]. However, some of the data were derived from experiments with murine monoclonal anti-DNA antibodies with uncertain clinical relevance in human lupus. Also, some studies employed solid-phase binding assays, which could introduce binding artifacts and conformational changes to the surface-bound antigens [64], and therefore, *in vitro* and experimental studies should be undertaken to confirm such findings. More recently, our group showed that human anti-dsDNA antibodies could bind to annexin II on the surface of human mesangial cells and induce changes in cell function [18].

## 3. Anti-dsDNA Antibody Binding to Kidney Cells and Renal Injury

Renal injury in lupus nephritis is initiated by the deposition of autoantibodies and/or immune complexes in the renal compartments. Downstream pathogenic effector mechanisms include activation of the complement and coagulation cascades, infiltration of acute and chronic inflammatory cells, and induction of mediators of inflammation or fibrosis from resident kidney cells and infiltrating cells. Polyclonal B-cell activation and autoantigen-driven expansion of autoreactive B cells result in the increased production of polyclonal anti-dsDNA antibodies in lupus patients and their deposition in sites of injury [65, 66]. Morphologic changes in the kidney are variable as reflected by the spectrum of pathological changes in lupus nephritis [67]. Previous studies have demonstrated heterogeneity in the molecular pathogenesis between patients with lupus nephritis [68]. Depending on the type, duration, and severity of lupus nephritis, immune deposits can be found in the mesangium, subendothelial, subepithelial, and tubulo-interstitial regions [67]. Deposition of cationic immune deposits in the mesangial or subendothelial compartments can initiate the recruitment of inflammatory cells and the activation of resident mesangial and endothelial cells [56]. Immune deposition in the subepithelial area is associated with podocyte injury and proteinuria, while the GBM acts as a barrier for leukocyte infiltration [56]. Irrespective of the site of initial or predominant injury, downstream events such as deposition of extracellular matrix and renal scarring constitute a final common pathway.

Data from *in vitro* studies have demonstrated that anti-dsDNA antibodies can bind to mesangial cells, glomerular epithelial cells (podocytes), endothelial cells, and proximal tubular epithelial cells [4, 5, 69–72], and that such binding led to functional changes in these cells [5, 18, 72–77]. The following discussion will focus on the results to date on the interaction between anti-dsDNA antibodies and resident renal cells in the context of the pathogenesis of lupus nephritis (Table 2).

### 3.1. Anti-DNA Antibodies and Mesangial Cells

Mesangial cells constitute up to 40% of the total cells in the glomerulus and are situated centrally within the glomerulus [78]. They are contractile and have morphological and functional properties similar to smooth muscle cells. Mesangial cells are able to synthesize a plethora of cytokines, growth factors, and matrix proteins which, together with their contractile property, provide structural support to the capillary loops and contribute to kidney homeostasis. Mesangial cells contribute to the synthesis and remodeling of extracellular matrix, which together with the cells constitute the mesangium. Qualitative and quantitative changes to the mesangial matrix can have a profound effect on mesangial cell function and behavior [78, 79]. As a corollary, these properties also explain the pathophysiology that follows mesangial cell injury.

Deposition of immunoglobulins and activation of complement within the mesangium is a cardinal feature in lupus nephritis, while complement activation plays an important role in the pathogenesis of different types of glomerular diseases [80]. Fenton et al. demonstrated that deposition of immune complexes in the mesangium of NZB/W F1 mice during the early phase of disease was accompanied by the appearance of anti-dsDNA antibodies, which preceded the downregulation of DNase 1 mRNA and activity [81]. In this regard, reduced renal expression of DNase 1 in lupus-prone mice is thought to be a mechanism for reduced fragmentation and clearance of chromatin material [53]. There is evidence that mesangial cells can synthesize C3, which is increased when the cells are incubated with immune complexes [82]. Mesangial cells thus have the potential to contribute to complement activation in the kidney and to complement-mediated injury in the mesangium.

The administration of anti-dsDNA antibodies to either predisease NZB/W F1 or BALB/c mice results in their deposition in the glomerulus, including the mesangium, through indirect chromatin-mediated or direct cross-reactive binding [13, 18, 83]. We and others have demonstrated that anti-dsDNA antibodies can bind to mesangial cells through chromatin material [69] or through the direct interaction with cross-reactive antigens such as *α*-actinin, annexin II, or ribosomal P protein, and that such binding may or may not be dependent on the Fc portion [16–18, 38, 84]. The functional consequences of this interaction include increased mesangial proliferation, apoptosis, activation of the PKC and MAPK signaling pathways, and increased synthesis of proinflammatory cytokines and profibrotic mediators such as hyaluronan, IL-1*β*, IL-6, TNF-*α*, TGF-*β*1, and fibronectin [18, 28, 72, 75, 76, 84–86].

The data that *α*-actinin can mediate the binding of anti-dsDNA antibodies to mesangial cells is intriguing, since *α*-actinin is an intracellular constituent of the mesangial cytoskeleton. *α*-actinin is present in multiple subcellular regions such as cell-cell and cell-matrix contact sites, in addition to cellular protrusions and lamellipodia [87, 88]. It is thus possible that part of the *α*-actinin molecule may extrude through the plasma membrane of mesangial cells to permit its binding with anti-dsDNA antibodies, although this needs to be confirmed by further studies. We have previously demonstrated that *α*-actinin expression is increased within the mesangium of patients with proliferative renal diseases [88]. Consistent with our finding, Zhao et al. also observed increased *α*-actinin expression in mesangial cells isolated from MRL/lpr mice [89], thereby suggesting increased availability of *α*-actinin for anti-dsDNA antibody binding. The pathogenic role of *α*-actinin as a cross-reactive antigen has recently been questioned by Mjelle et al. who demonstrated that anti-dsDNA antibodies did not colocalize with *α*-actinin in kidneys obtained from NZB/W F1 mice, but instead bound to glomerular structures containing extracellular nucleosomes [90].

Annexin II is a calcium-dependent, phospholipid binding protein that is expressed in various organs including the kidney and can exist either as a monomer, heterodimer, or heterotetramer [91]. Annexin II is present within the cytoplasm and on the plasma membrane of various cells [91], and its translocation from the cytoplasm to the plasma membrane is increased following its phosphorylation that can be induced by various cytokines and growth factors such as IFGF and EGF or heat stress [92, 93]. It functions as a plasminogen receptor, thus regulating fibrin homeostasis and angiogenesis and also membrane trafficking [94]. Autoantibodies to annexin II are detected in patients with antiphospholipid syndrome, and annexin II has been shown to activate endothelial cells following their exposure to antiphospholipid antibodies [95]. We have recently demonstrated that annexin II is a cross-reactive antigen on the surface of human mesangial cells that mediates the binding of human polyclonal anti-dsDNA antibodies [18]. We further demonstrated that following the binding of anti-dsDNA antibodies to annexin II, the antibodies were internalized and translocated to the cytoplasm and nucleus in a time- and temperature-dependent manner [18]. The ability of anti-dsDNA antibodies to penetrate live cells was first observed by Alarcon-Segovia and Llorente in human mononuclear cells [96], and subsequently by Yanase et al. in rat H35 hepatoma cells [97, 98]. It is noteworthy that entry of antibodies into cells is not unique to anti-dsDNA antibodies, since this phenomenon has also been observed with autoantibodies against nuclear ribonucleoprotein, ribosomal P protein, La, and Ro in human and animal cells [99–102].

Data from our laboratory showed that binding of anti-dsDNA antibodies to annexin II on mesangial cell surface induced annexin II synthesis in the cells, and the latter was mediated through the activation of p38 MAPK, JNK, and AKT/PI3K signaling pathways. There was also a concomitant increase in cell proliferation, induction of IL-1*β*, TNF-*α*, IL-6, and hyaluronan secretion, activation of the PKC signaling pathway, and upregulation of TGF-*β*1 and fibronectin synthesis [18, 75, 76]. These findings thus propose a new paradigm by which anti-dsDNA antibodies contribute to progressive inflammatory and fibrotic processes in the pathogenesis of lupus nephritis. The effect on IL-6 is worth highlighting, since this cytokine has been shown to increase mesangial cell proliferation and exacerbate glomerulonephritis [103]. Our results from animal experiments and human renal biopsies also showed that annexin II expression was increased in the mesangium and GBM of NZB/W F1 mice and patients with active lupus nephritis, and it colocalized with IgG and C3 deposition [18]. Intercepting the interaction between anti-dsDNA antibodies and annexin II on mesangial cells may therefore be a potential novel approach for the treatment of lupus nephritis.

The mechanism(s) through which autoantibodies are internalized, and the functional consequence of this process, have yet to be fully elucidated, but it has been suggested that some of these autoantibodies may be internalized through an Fc receptor-dependent mechanism, which was associated with cellular changes such as cytotoxicity and apoptosis [104]. Internalization and nuclear localization of anti-dsDNA antibodies may also be dependent on their polyreactivity and the presence of nuclear localizing motifs in the CDR3 region of the heavy chain [105]. It is possible that these autoantibodies are transported intracellularly via clathrin-associated vesicles or are accompanied by chaperones [106]. The administration of murine anti-dsDNA antibodies to non-autoimmune mice resulted in their localization in the cell nuclei of many organs including the kidney, and this was associated with glomerular hypercellularity, increased collagen expression in the mesangial matrix, and proteinuria [107]. However, cellular entry and localization of these anti-dsDNA antibodies were shown to be dependent on the antigen-binding region of the molecule but not mediated through the Fc-receptor, although the role of Fc-mediated inflammation in the mesangium through other pathways cannot be excluded [107, 108].

### 3.2. Anti-DNA Antibodies and Endothelial Cells

The glomerular capillary endothelium differs from other endothelial cells in that they are flattened and highly fenestrated [109]. Endothelial cell activation and injury is a common occurrence in various immune-mediated glomerular diseases where there is complement activation in the subendothelial space, as observed in severe proliferative lupus nephritis [67, 110]. Activation of glomerular endothelial cells results in the upregulation of adhesion molecules [111], which serves to stabilize the adhesion of infiltrating leukocytes to the sub-endothelial and mesangial regions during immune-mediated renal injury.

Anti-endothelial cell antibodies have been detected in the serum of a high proportion of lupus patients, especially during active disease [112–118]. Serum levels of anti-endothelial cell antibodies correlate with the severity of lupus nephritis and serological evidence of endothelial dysfunction [116, 119]. Anti-endothelial cell antibodies have also been shown to induce glomerulonephritis in normal rabbits [120]. Fujii et al. observed that intraperitoneal injection of 17H8a, a hybridoma clone derived from MRL/lpr mice, into SCID mice resulted in the deposition of 17H8a antibodies in the subendothelium and the formation of glomerular lesions similar to those in lupus-prone mice [121]. Furthermore, the 17H8a antibodies could be internalized by human umbilical vein endothelial cells (HUVECs) and glomerular endothelial cells through a mechanism that was mediated by fibronectin and actin polymerization [121]. It was not reported whether 17H8a antibodies were reactive towards dsDNA.

Subsets of murine monoclonal anti-dsDNA antibodies can bind to HUVEC indirectly through chromatin material, indicating that anti-dsDNA antibodies contribute to the repertoire of anti-endothelial cell antibodies [69, 70, 117]. We have previously reported that, in the presence of DNA, some murine anti-dsDNA antibodies are able to bind to an HUVEC plasma membrane protein with an M.W. of 46 kDa, and the ability of DNA to bind to the surface of HUVEC was increased in the presence of IL-1*α* or TNF-*α* [70]. Histones could also facilitate the binding of murine monoclonal anti-dsDNA antibodies and DNA to HUVEC, and the degree of binding was influenced by the relative concentrations of antibody, DNA, and histones [69]. In the human setting, polyclonal anti-dsDNA antibodies isolated from different patients with lupus nephritis could bind to HUVEC through two distinct mechanisms. Human polyclonal anti-dsDNA antibodies that required DNA to bind to HUVEC also bound to a protein of M.W. 46 kDa, whereas direct cross-reactive binding was mediated through membrane proteins with M.W. of 30–35, 44, 68, 110, and 180 kDa [122]. Incubation of HUVEC with human polyclonal anti-dsDNA antibodies induced the expression of VCAM-1, ICAM-1, and von Willebrand factor, which was associated with increased expression of IL-1, IL-6, and IL-8, which had been shown to play an important role in the inflammatory processes in lupus nephritis [73, 74, 123, 124].

Accumulating evidence suggests that type I interferons (IFN) such as IFN-*α* and IFN-*γ* play an important role in the pathogenesis of lupus nephritis [125, 126]. These proinflammatory peptides are synthesized by both infiltrating and resident renal cells including glomerular endothelial cells [127]. Recent studies have demonstrated that type I IFN synthesized by resident renal cells promoted end-organ disease in an experimental model of autoantibody-mediated glomerulonephritis [128]. It is possible that anti-dsDNA antibodies may also induce synthesis of IFN-*α* and IFN-*γ* in endothelial cells and other intrinsic renal cells to mediate downstream inflammatory processes although further studies are warranted to confirm this.

### 3.3. Anti-DNA Antibodies and Proximal Renal Tubular Epithelial Cells

Approximately 70% of lupus nephritis patients have demonstrable immune aggregates along the renal tubular basement membrane. The tubulo-interstitium occupies up to 90% of the kidney volume. Variable degrees of tubulo-interstitial inflammation and fibrosis are found in practically all forms of chronic progressive renal diseases regardless of the inciting injury, including those which start off as a predominantly glomerular disease, and the severity of tubulo-interstitial changes inversely correlates with renal prognosis [129, 130]. Proximal renal tubular epithelial cells constitute the predominant cell type in the tubulo-interstitium. These cells are responsible for solute transport and reabsorption. They have the ability to synthesize growth factors and matrix proteins and have a high proliferative potential and thus are important in the regeneration of the tubular epithelium in response to acute tubular injury. Upon stimulation by proinflammatory or profibrotic mediators, proximal renal tubular epithelial cells exhibit phenotypic alterations and undergo epithelial-to-mesenchymal transdifferentiation (EMT) [131]. EMT is characterized by the loss of epithelial cell adhesion, cell activation with actin reorganization and *de novo* synthesis of *α*-smooth muscle actin, disruption of the underlying basement membrane, and increased cell migration and invasion [132–134]. There is compelling evidence that the presence of myofibroblasts predicts progressive fibrosis in animal and human renal diseases [135–140].

We have demonstrated that the deposition of immune complexes in the tubulo-interstitium correlated with circulating anti-dsDNA antibody levels, tubulo-interstitial expression of IL-6, and tubulo-interstitial abnormalities that included tubular atrophy, inflammatory cell infiltration, and interstitial fibrosis [5]. The level of tubulo-interstitial IL-6 expression, predominantly contributed by the proximal tubular epithelial cells, correlated with the infiltration of immune cells into the tubulo-interstitium.

HK-2 cells are normal proximal renal tubular epithelial cells that have been immortalized by transduction with the human papilloma virus [141]. We have demonstrated that anti-dsDNA antibodies from patients with lupus nephritis induced phenotypic changes in HK-2 cells that were analogous to epithelial cells undergoing EMT [5]. We have also demonstrated that anti-dsDNA antibodies, especially those derived from patients during active disease, could induce IL-6 secretion in HK-2 cells [5]. Depending on the disease status, the induction of IL-6 secretion in HK-2 cells by anti-dsDNA antibodies can be through distinct mechanisms. During remission, anti-dsDNA antibodies induced IL-6 secretion either directly or were mediated through IL-1*β*. In contrast, anti-dsDNA antibodies isolated from patients with active disease induced IL-6 secretion through the prior induction of both IL-1*β* and TNF-*α* [5]. The heterogeneity may be related to distinct properties of different clones of anti-dsDNA antibodies. Mediators secreted by mesangial cells and HK-2 cells upon stimulation with anti-dsDNA antibodies can induce IL-6 secretion in the other cell types, indicating that there could be bidirectional communication or crosstalk between the glomerulus and tubulo-interstitium [5]. Consistent with our findings, Ronda et al. also observed that immunoglobulins isolated from the sera of patients with SLE could induce IL-6 secretion in proximal renal tubular epithelial cells, and this was accompanied by the activation of the ERK signaling pathway [142].

Koren et al. have reported that murine and human anti-dsDNA antibodies cross-reacted with A and D snRNP proteins in porcine proximal tubular epithelial cells (PK15 cells). Of the two murine monoclonal antibodies tested that were derived from NZB/W F1 mice, one anti-dsDNA antibody was internalized and localized in the cytoplasm and nuclei of cells, while the second murine monoclonal antibody remained at the cell surface and was not internalized. The concomitant addition of complement and either murine or human anti-dsDNA antibodies to PK15 cells resulted in cell lysis, which was more prominent with the subset of anti-dsDNA antibodies that were not internalized [143]. Zack et al. demonstrated that the murine anti-dsDNA antibody mAb 3E10 could bind to renal tubules in normal human renal tissue, and intraperitoneal injection of mAb 3E10 into normal BALB/c mice primed with pristane resulted in antibody binding to the plasma membrane of proximal renal tubular epithelial cells and their subsequent internalization and translocation into the nucleus [71]. This binding was dependent on DNA and the Fab portion of the antibody [71]. These studies have thus begun to shed light on the pathogenic pathways through which anti-dsDNA antibodies can induce tubulo-interstitial injury through their interaction with proximal renal tubular epithelial cells.

### 3.4. Anti-DNA Antibodies and Podocytes

Podocytes are highly differentiated epithelial cells that are found on the outer surface of the glomerular capillary tuft and serve as the final barrier to urinary protein loss by the formation of foot processes and interposed slit diaphragms [144]. We and others have demonstrated that anti-dsDNA antibodies can bind to podocytes *in vitro* and *in vivo* [4, 90, 145]. The administration of a human monoclonal anti-dsDNA/anti-*α*-actinin antibody to SCID mice resulted in their subepithelial and subendothelial deposition, which was associated with widespread, segmental effacement of podocyte foot processes and proteinuria [145]. Although it is tempting to speculate that anti-dsDNA antibodies may bind to podocytes through *α*-actinin, studies by Mjelle et al. suggest otherwise [90]. These researchers proposed that nucleosomes mediated the binding of anti-dsDNA antibodies to glomerular structures* in vivo* [90]. Subepithelial immune deposits along the peripheral capillary loops is a cardinal feature in patients with class V lupus nephritis, which results in podocyte hypertrophy, their increased synthesis of matrix proteins, and subsequent thickening of the GBM [146].

## 4. The Role of Toll-Like Receptors in the Pathogenesis of Lupus Nephritis

TLRs are a class of innate immune receptors that regulate inflammatory and immune responses. They are essential for the induction of adaptive immune responses against microbial infection [147]. Although they are predominantly expressed on leukocytes, resident renal cells also express distinct members of the TLR family depending on the cell type [148]. Accumulating evidence suggests that TLRs contribute to the pathogenesis of lupus nephritis, where nonmicrobial, host-derived nucleic acids activate TLRs and exacerbate disease manifestations [149–151]. TLR-9 recognizes hypomethylated CpG-containing DNA motifs including those of mammalian origin released from injured or stress-induced cells [149, 152]. Activation of TLR-9 by DNA-containing immune complexes is mediated through high-mobility group box proteins and receptor for advanced glycation end-products [149], and once activated, it augments cytokine production in dendritic cells [149, 153]. TLR-9 activation also regulates the production of anti-dsDNA antibodies in lupus-prone mice [60, 154]. The expression of TLR-9 in resident renal cells is arguable since some researchers have shown TLR-9 to localize solely on infiltrating cells [155], whilst other researchers have observed increased TLR-9 expression in tubular epithelial cells and glomerular cells during active lupus nephritis [156, 157]. Increased tubular expression of TLR-9 correlates with proteinuria and tubulo-interstitial injury in lupus patients, whereas increased glomerular TLR-9 expression is associated with a higher activity index [156, 157]. Inhibition of TLR-9 signaling in lupus-prone mice attenuates the development of glomerulonephritis [158] although its pathogenic role in the development of lupus nephritis has recently been questioned [159]. Mesangial and tubular epithelial cells also express TLR1-4 and TLR-6 [148, 155], but their role in the pathogenesis of lupus nephritis remains to be defined.

## 5. Conclusion

Renal involvement is a major cause of morbidity and mortality in SLE. Pathological manifestations in lupus nephritis are diverse, initiated by the deposition of immunoglobulins and formation of immune complexes in the glomerular and tubulo-interstitial compartments of the kidney. There is emerging evidence that the interaction between anti-dsDNA antibodies and resident kidney cells, notably mesangial cells, proximal renal tubular epithelial cells, glomerular endothelial cells, and possibly podocytes, plays a significant role in disease pathogenesis. Cell surface binding followed by translocation of antibodies to the cytoplasm and/or nucleus precedes the induction of proinflammatory and profibrotic pathways. Distinct mechanisms may apply to different subsets of antibodies, and at different phases of disease. Not only does the elucidation of these processes provide researchers with a better understanding of the role of anti-dsDNA antibodies in pathogenesis, but also it offers potential novel approaches for disease intervention.

## Figures and Tables

**Table 1:** Autoantibodies with pathogenic potential in patients with lupus nephritis.

Autoantibodies	Prevalence (%)	Binding to kidney structure/resident renal cells	References
Anti-dsDNA	70–96	GBM	[29–31]
		Mesangial cells	
		Glomerular epithelial cells	
		Glomerular endothelial cells	
		Proximal tubular epithelial cells	
Anti-nucleosome	60–90	GBM	[29, 31, 32]
		Mesangial cells	
		Glomerular epithelial cells	
		Glomerular endothelial cells	
Anti-Ro	25–44	GBM	[29, 33]
Anti-Smith	10–60	GBM	[29, 30, 33]
Anti-C1q	40–97	GBM	[29, 31, 34, 35]
		Glomerulus	
		Tubular basement membrane	
Anti-*α*-actinin	20	Glomerulus	[16, 17, 29, 36]
		Mesangial cells	
		Podocytes	
Anti-annexin II	32–65	Glomerulus	[18]
		Mesangial cells	
Anti-ribosomal P protein	75	Glomerulus	[37, 38]
		Mesangial cells	

**Table 2:** Binding of anti-dsDNA antibodies to resident renal cells and the effect on cellular functions.

	Mesangial cells	Endothelial cells	Proximal renal tubular epithelial cells
Mechanism of binding	Indirect binding through	Indirect binding through	Indirect binding through
	DNA, histones, and nucleosomes	DNA, histones, and nucleosomes	DNA
	Cross-reactive binding to	Cross-reactive binding to	Cross-reactive binding to
	heparan sulfate	hevin	A and D snRNP proteins
	ribosomal P protein	unidentified proteins with M.W.	
	laminin	of 30–35, 44, 68, 110, and 180 kDa	
	*α*-actinin		
	annexin II		
Internalization of anti-dsDNA	Occurs after binding to annexin II	Occurs after binding to fibronectin	Occurs after binding to
antibodies into resident renal cells			unidentified protein(s)
Effect on cell proliferation	Increase	Increase	Increase
Induction of apoptosis	Yes	Yes	Yes
Effect on cell viability	Decrease	Decrease	Decrease
Effect on inflammation	Increased synthesis of:	Increased synthesis of:	Increased synthesis of:
	IL-1*β*, IL-6, and TNF-*α*	IL-1*β*, IL-6, and IL-8	IL-1*β*
	hyaluronan	adhesion molecules	IL-6
		von Willebrand factor	TNF-*α*
Effect on fibrosis	Activation of PKC-*α*, -*β*I,	Increased gene expression	Induced epithelial-to
	and *β*II signaling pathways	of TGF-*β*1	mesenchymal
	and increased synthesis of		transdifferentiation
	TGF-*β*1 and fibronectin		
